# Health behaviors in school-aged children: global trends in education, socioeconomic status, and adolescent health

**DOI:** 10.3389/fpubh.2025.1514386

**Published:** 2025-06-03

**Authors:** Mastour Saeed Alshahrani, Vamsi Krishna Gannamaneni, Venkata Nagaraj Kakaraparthi, Khalid A. Alahmari, Batool Alkhamis, Ravi Shankar Reddy, Jaya Shanker Tedla, Snehil Dixit, Lalitha Kakaraparthi, Gopal Nambi

**Affiliations:** ^1^Department of Medical Rehabilitation Sciences, King Khalid University, Abha, Saudi Arabia; ^2^Department of Physiotherapy, University of Hail, Hail, Saudi Arabia; ^3^Department of Physical Therapy and Health Rehabilitation, Prince Sattam Bin Abdulaziz University, Al-Kharj, Saudi Arabia

**Keywords:** adolescent health, cultural health paradigms, health surveys, social determinants of health, health policies

## Abstract

The study explores global trends in adolescent health using data from the health behavior in school-aged children (HBSC) study, focusing on the role of education, socioeconomic status, and school-based health programs. It examines cross-country comparisons to assess disparities in adolescent wellbeing and health behaviors. Findings highlight that nations with comprehensive public health policies report lower adolescent obesity rates and better mental wellbeing, whereas regions with limited resources, experience greater health inequalities. The key factors contributing to these disparities include economic conditions, access to healthcare, and variations in school health programs. The study underscores the need for evidence-based, culturally adaptable interventions to improve adolescent health outcomes. By analyzing current policies and identifying gaps, this review aims to guide future public health strategies toward reducing inequalities and strengthening school-based health initiatives worldwide.

## Introduction

The Health Behavior in School-aged Children (HBSC) study, initiated in 1983, is the largest study on child and adolescent health and one of the utmost eminent sources of data for the WHO’s global health monitoring system (1). The Helsinki-based international research project coordinated by the WHO analytically collected statistics on the health behaviors, lifestyles, and wellbeing of children across various countries (2–4). For decades, HBSC has examined the influence of social, economic, and environmental factors on adolescent health (5). These encompassed a variety of structural and practical variables, such as differences in the educational systems where fieldwork was carried out, adherence to a standard research protocol, problems with language and translation, and disparities in statistical capacities within nations.

The HBSC study has been widely regarded as a crucial tool for understanding adolescent health and wellbeing. However, like any large-scale cross-national study, it is not without controversies or different schools of thought.

Here are some key debates, criticism point of view and counterpoint perspectives surrounding the HBSC study:

### Cultural and socioeconomic contexts

#### Debate and critique

National-level data may be incomprehensible and may provide significant regional disparities, leading to generalized health policies. In Canada, for example, 24% of adolescents state low life satisfaction, but rural areas experience greater dissatisfaction levels than urban centers (6). Similarly, in Mexico, more than 32% of adolescents report low life satisfaction, with prominent variances between richer urban regions and poorer rural areas (7). In Mozambique, where 40% of adolescents report low life satisfaction, national economic uncertainty and limited mental health sources likely influence the issue (8).

#### Counterpoint

However, HBSC allows between-country comparisons and is therefore useful to illustrate the influence of local contexts in specifying health behavior determinants that should address country specificities for future initiatives targeting adolescent wellbeing.

### Measurement and validity concerns

#### Debate and critique

The HBSC depend comprehensively on self-reported statistics, raising interests about reliability and social suitability bias. For instance, self-reported alcohol consumption differs considerably, 16% of 15-year-olds in Norway conveyed drinking alcohol in previous years (9), compared to 38% in Hungary (10) and 8% in Nepal (11). Cultural norms perhaps influence these changes, with Hungarian adolescents more oriented to report drinking due to superior societal acceptance, while Nepalese adolescents may underreport due to religious and social humiliation.

#### Counterpoint

The HBSC uses confidentiality the protections to decrease social appropriateness, bias, and enormous sample sizes to help to balance individual reporting miscalculations. Despite potential biases, the study provides constructive data for identifying public health concerns and guiding policy interventions.

### Focus on school-based interventions

#### Debate and critique

Focusing primarily on schools as sites for intervention may ignore other important influences on adolescent health, such as family dynamics or community environments. For example, in high-income country like Germany, 17% of adolescents reported being bullied at school (12), while in middle-income Brazil, the figure is 26% (13). In a low-income country like Ethiopia, 21% of adolescents reported being bullied at school (14). While schools remain critical environments for intervention, focusing solely on school settings may miss other important factors, such as family dynamics or community issues. For instance, in Brazil, social inequality and gang violence might play a significant role in bullying, while in Ethiopia, economic hardship and family stress might contribute to bullying behaviors, extending beyond the classroom environment.

#### Counterpoint

The schools remain important settings for intervention because they provide structured environments where policies (such as anti-bullying programs) can be implemented effectively. The HBSC’s emphasis on schools can guide policymakers in creating interventions that target students while also considering external influences.

### Scope of data and health indicators

#### Debate and critique

Critics argue that the HBSC should expand its scope to include emerging health issues like the impact of digital technologies on adolescent wellbeing.

For example, in high-income Finland, 32% of adolescents report spending more than 4 h per day on screens (e.g., smartphones or computers) (15). In middle-income Mexico, this number is higher, with 44% of adolescents reporting excessive screen time (16). In a low-income country like Uganda, 29% of adolescents report spending too much time on screens (17). Although the HBSC tracks screen time, it may not fully account for the complex impacts of digital culture and social media on adolescent health, especially in countries like Mexico where cyberbullying and social media-related anxiety are growing concerns.

#### Counterpoint

The HBSC’s optional modules allow countries to investigate emerging trends like social media use and its health impacts. In Finland, for instance, additional questions on cyberbullying have been added to better understand the digital landscape and its effects on youth.

### Policy implications and real-world impact

#### Debate and critique

While the HBSC provides valuable data, translating it into effective public health policies can be challenging, especially in countries with varying resources.

For example, in high-income country like Sweden, 24% of adolescents meet the World Health Organization’s (WHO) physical activity recommendations of at least 60 min of daily exercise (18). Sweden has successfully implemented national policies promoting physical activity through schools and public health campaigns (19). In middle-income country like Bulgaria, 18% of adolescents meet the recommendation, reflecting lower investment in public health infrastructure and fewer school-based programs (20, 21). In a low-income country like Haiti, only 10% of adolescents meet the daily activity recommendation (22). Haiti faces significant challenges, such as limited funding for school sports programs and a lack of public recreational facilities, making it difficult to translate HBSC findings into policy improvements (23).

Translating HBSC findings into actionable policies can be difficult, especially in countries with limited public health resources, like Haiti. Implementing wide-reaching programs to encourage physical activity may require external funding or international partnerships.

#### Counterpoint

The HBSC provides governments with the evidence needed to advocate for policies promoting physical activity. Even in low-income countries the data can be used to attract support from international organizations or guide efforts to improve health outcomes despite resource limitations.

These examples illustrate how high, middle, and low-income countries experience varying challenges and opportunities in adolescent health, based on HBSC data. Cultural, economic, and policy contexts heavily influence health behaviors and outcomes, and the HBSC helps identify these differences to inform targeted interventions across different regions.

## Multidimensional approach to health: adolescent/school oriented view

The HBSC study is unique in its multi-dimensional perspective on adolescent health (24). Typical health-related surveys commonly evaluate only one behavior such as smoking or dietary habit. In contrast, various items were measured by HBSC including mental health problems (high depressive mood, stress and headache), obesity indexes substance use, sexual behavior indicators and knowledge on sexually transmittable infections as well early complications of having offspring with adolescent’s pregnancy rate (25). By using this overarching perspective, investigators are then able to assess not only individual behaviors but also at the interplay between these behaviors and a plethora of social determinants on health (26). For example, there is also research to suggest that dietary habits in young people are linked with levels of physical activity and mental health outcomes (27).

## Objectives of the study

This review aims to:

To critically examine the scope, methodology, and evolution of the HBSC study across different survey cycles and participating countries.

To assess the contribution of HBSC data to the understanding of key adolescent health behaviors, including mental health, substance use, screen time, and dietary habits.

To compare and contrast international and country-specific findings from official HBSC reports and peer-reviewed literature to highlight consistencies, gaps, and divergences.

To identify methodological challenges and data validity concerns in existing HBSC-related research and reporting practices.

To highlight research gaps and propose future directions for leveraging HBSC data in policy development, school-based interventions, and global health strategies.

### Methodology

This study is a narrative review that synthesizes results from existing research on adolescent health behaviors, focusing on education, socioeconomic status, and wellbeing.

### Data collection and selection criteria

Data was collected from peer-reviewed journal articles, reports from international organizations (e.g., WHO, HBSC), and government health surveys published from 1983 until 2024. Literature was categorized through systematic searches in MEDLINE, EMBASE, CINAHL, LILACS, SCIELO, DOAJ, PubMed, PEDro, Saudi digital library, NHS EED, PROSPERO, Google Scholar, Scopus, and Web of Science using keywords such as “adolescent health,” “socioeconomic status,” “education and health behaviors,” and “school-based interventions.”

Studies were included mainly which focused on school-aged children (11–15 years old), provided cross-national comparisons of adolescent health indicators, and examined socioeconomic influences on adolescent wellbeing.

Exclusion criteria included articles with limited regional focus or lacking relevance to adolescent health behaviors.

#### Data analysis and synthesis

Extracted information was categorized under key themes: (1) socioeconomic disparities, (2) education and school health programs, (3) mental health trends, (4) obesity and physical activity, and (5) substance use.

A comparative thematic analysis was performed to identify patterns, disparities, and policy implications across different countries. The findings were critically evaluated to highlight gaps in research and policy recommendations for improving adolescent health outcomes. This approach ensures a structured synthesis of evidence, enabling meaningful cross-national comparisons without statistical testing.

In this study, we conducted cross-national comparisons using data from the Health Behavior in school-aged children (HBSC) study, which includes over 50 participating countries. The comparisons focused on adolescent health indicators such as obesity rates, mental health outcomes, physical activity levels, and school-based health interventions. Specifically, we examined countries with well-established public health programs (e.g., Norway, Sweden, Denmark), nations facing higher social inequalities and health disparities (e.g., Romania, Lithuania, Poland), and middle-income countries with diverse adolescent health profiles (e.g., Mexico, Bulgaria, Turkey). The selection of countries was based on available HBSC data and their relevance to the study’s objectives.

By specifying the countries and the criteria for comparison, this study aims to provide a clearer understanding of how national policies and socioeconomic contexts influence adolescent health outcomes globally.

Countries with comprehensive school-based health policies, such as Norway, Sweden, and Denmark, report lower adolescent obesity rates and enhanced mental health outcomes due to well-established nutrition programs, physical activity initiatives, and mental health support services. In contrast, Romania, Lithuania, and Poland experience higher adolescent health disparities, largely driven by socioeconomic inequalities and limited access to school-based health services. Additionally, middle-income countries like Mexico, Bulgaria, and Turkey show varied adolescent health outcomes, influenced by differing levels of investment in health promotion and school-based interventions.

For example, in countries, where strong public health policies are in place, adolescent obesity rates remain relatively low, with Norway and Sweden reporting rates of 8 and 9%, respectively. In contrast, Romania and Lithuania, where socioeconomic disparities limit access to school-based health interventions, report higher obesity rates of 32 and 28%. Similarly, mental health concerns are more prevalent in European nations, with Estonia, Lithuania, and Romania reporting 25, 27, and 30% of adolescents experiencing anxiety and depression, respectively, compared to 12% in Finland. These variations highlight the role of national policies in shaping adolescent health outcomes (12, 28–32). The comparative nature of the HBSC study also provides an informative model for public health responses to adolescent wellbeing, worldwide (1, 33–36) (Table 1).

**Table 1:** Adolescent health related concerns across different countries.

Country	Mental health issues (%)	Obesity rate (%)	Substance abuse (%)	Sexual health education (%)	Teen pregnancy rate (%)
Australia	20	25	18	90	10
Austria	18	14	15	85	7
Belgium	22	20	16	88	9
Canada	15	30	14	92	8
Czech Republic	19	27	13	75	6
Denmark	16	10	11	95	5
Estonia	25	16	19	80	12
Finland	12	11	8	90	4
France	21	19	20	85	10
Germany	18	17	17	86	9
Greece	20	24	14	80	11
Hungary	23	30	16	70	13
Iceland	14	8	7	92	3
Ireland	19	25	12	87	10
Italy	18	20	15	83	8
Latvia	24	22	18	76	14
Lithuania	27	28	19	72	15
Luxembourg	16	13	10	88	6
Malta	21	18	16	75	12
Netherlands	15	10	9	95	4
New Zealand	17	26	13	91	11
Norway	13	9	8	94	5
Poland	26	29	21	73	16
Portugal	19	24	12	82	9
Romania	30	32	20	65	18
Slovakia	22	26	15	70	14
Slovenia	16	15	11	78	8
Spain	20	23	17	84	12
Sweden	12	11	6	95	3
Switzerland	15	9	10	90	5
United Kingdom	19	29	20	88	15
United States	24	35	23	80	17

## Understanding adolescent health (key observations)

### Mental health issues

Estonia (25%), Lithuania (27%), and Romania (30%) reported the highest proportions of 11–15 year olds with mental health difficulties such as anxiety and depression are reported (37). These countries may need to boost their provision of mental health services and interventions for young people. Iceland and Norway have the lowest percentages (14% for both) followed by Finland with 12%, indicating better mental health access to support the intensity of psychiatric follow-up in due to socioeconomic background/counselor activity/surrounding social environment (31, 38).

### Obesity rates

Top ranked in the world, for obesity rates of adolescent obesity, some of the highest worldwide with examples including United States (35%), Romania (32%) and Lithuania both at 28% highlighting challenges to dietary habits and physical activity (29, 39). Iceland (8%), Norway (9%), and Switzerland (9%) have significantly lower obesity rates, likely reflecting better access to healthy foods and more active behaviors in daily activities of life (40, 41).

Substance abuse in adolescents is higher among the United States and Poland (23%), Romania, 20%, making for a call up on prevention programs and public education to combat drug usage (42). Countries like Sweden (6%), Iceland (7%), and Finland (8%) report the lowest substance abuse rates, possibly due to comprehensive school-based prevention programs (5, 43).

### Sexual health education

Unequivocally the top three countries for sexual health education are Denmark, Sweden and Netherlands with 95% each, all benefiting from established school-based programs focusing on teaching adolescents safe practices. In Romania, 65% and in Hungary only 70% of adolescents reported having had information on sexual health which points toward an important gap concerning a key topic influencing adolescent wellbeing (44–46).

### Teen pregnancy rates

Romania (18%), United States (17%), and Poland (16%) report the highest rates of teen pregnancy, highlighting the need for more effective sexual education and access to reproductive health services. Iceland (3%), Sweden (3%), and Finland (4%) have the lowest teen pregnancy rates, suggesting strong sexual education and healthcare systems (46–48).

## Cross-national comparisons and global insights

One of the HBSC study’s defining features is its international range. The survey has been conducted in more than 50 countries and regions. Health behaviors and outcome differences across countries can be readily examined within this cross-national framework. For instance, studies have shown that different countries eat and exercise differently because of their culture and government efforts around health issues. Such insights highlight the need for health interventions tailored to the adolescent population in various regions (49).

This aspect of this study revealed factors such as physical activity, fruit consumption, vegetable consumption, smoking, alcohol use, and bullying across various countries. Countries that promote health well, e.g., Norway and Sweden have shared their successes with countries with high rates of adolescent health problems such as obesity and mental illness. This joint effort has the potential to enhance worldwide initiatives aimed at enhancing adolescent health results through evidence-based policy creation (50–54) (Table 2).

**Table 2:** Cross-national comparisons: selected health indicators from HBSC study.

Country	Physical activity (%)	Fruit consumption (%)	Vegetable consumption (%)	Smoking (%)	Alcohol use (%)	Bullying (%)
Australia	55	65	45	13	23	15
Austria	70	60	55	17	30	20
Belgium	62	58	50	15	25	19
Canada	56	75	50	12	20	14
Czech Republic	67	50	40	20	18	16
Denmark	80	72	60	9	15	10
Estonia	64	55	42	19	28	17
Finland	75	80	70	8	14	11
France	60	65	57	25	32	18
Germany	65	66	54	19	21	13
Greece	50	58	55	15	29	22
Hungary	60	52	47	21	26	19
Iceland	77	82	66	10	12	9
Ireland	64	68	48	15	27	21
Italy	58	60	52	18	24	15
Latvia	63	50	40	23	31	24
Lithuania	70	55	45	21	29	26
Luxembourg	68	65	50	11	18	12
Malta	55	45	38	20	34	20
Netherlands	78	75	62	7	12	8
New Zealand	63	67	53	15	22	16
Norway	82	80	65	6	10	7
Poland	60	56	42	19	27	23
Portugal	61	54	48	14	26	15
Romania	57	52	45	18	30	19
Slovakia	62	53	41	20	25	20
Slovenia	66	59	48	17	24	18
Spain	65	70	60	14	20	14
Sweden	80	78	65	10	15	9
Switzerland	72	74	55	12	18	13
United Kingdom	60	68	50	20	25	15
United States	54	62	40	14	27	22

### Key findings from the cross-national comparisons regarding health indicators

#### Physical activity

In Norway, Sweden and Denmark over four in five, 11-to 15-year-olds report doing physical activity (more than 80%) which is among the highest levels in European countries reflecting the importance of active lifestyles and the importance of sports activities. Greece (50%), United States (54%), and Australia (55%) had the lowest, which could reflect the less emphasis on levels of physical education or activities (39, 55).

#### Fruit and vegetable consumption

Iceland and Norway has the highest rates (both 80% fruit consumption, overall half respondents eating either on a daily basis) implying good nutrition education or availability of healthy food. At the lowest end of both fruit and vegetable intake are Malta (45% fruit, 38% vegetables consumption) and Czech Republic (50% fruit, 40% vegetables consumption) representing potential gaps in promoting healthy eating behaviors among adolescents (56, 57).

#### Smoking and alcohol use

Norway (6% smoking, 10% alcohol routine) and Netherlands (7% smoking, 12% alcohol routine) have the lowest rates of teenage smoking and alcohol intake, likely reflecting effective prevention programs and firmer policies. Higher rates are reported in France (25% smoking, 32% alcohol use) and Latvia (23% smoking, 31% alcohol use), implying that more tailored interventions are necessary to reduce substance use should be implemented among adolescents (58–60).

#### Bullying

Netherlands (8%), Norway (7%), and Sweden (9%) show the lowest rates of bullying, likely because they have anti-bullying policies that are much more comprehensive and support systems. Latvia (24%), Lithuania (26%), and Poland (23%) have higher rates, pointing to current trials in focusing bullying in these regions (61, 62).

## Revising social inequalities to provide the equal adolescent health opportunities is imperative

First and foremost, the existence of simple physical health adolescence opportunities is essential to eliminate these inherent social differences. This study too identified family affluence, neighborhood safety and access to healthcare and education as some of the socio-economic factors related to health outcome inequalities within adolescents. Addressing these inequities demands system change that goes beyond health systems to schools, communities and the broader social policy environment. In other words, the study underscores schools as not only important for both health and learning adolescent effectively a concept I have long argued but also provide boundary conditions aspect of wellbeing of adolescents.

For example, states that have in combination invested both in national school-based health services (free healthy meals and mental health service/extracurricular programs) alongside social policies which target to the most unequal forms of inequality using an index tool-witnessed improved adolescents’ wellbeing (24, 52, 63–66) (Table 3).

**Table 3:** Social inequalities across different countries (HBSC studies).

Country	Socioeconomic status (%)	Access to healthcare (%)	Education inequality (%)	Family support (%)	Neighborhood safety (%)
Australia	15	90	18	80	88
Austria	20	85	16	77	80
Belgium	18	82	22	79	83
Canada	14	92	15	85	90
Czech Republic	22	75	20	70	75
Denmark	10	95	12	85	92
Estonia	25	72	24	65	78
Finland	12	90	10	88	95
France	19	85	18	81	84
Germany	16	87	17	80	82
Greece	27	70	25	65	70
Hungary	30	65	26	60	65
Iceland	12	94	11	90	92
Ireland	18	88	20	83	85
Italy	22	80	21	76	78
Latvia	28	68	26	60	73
Lithuania	30	64	28	58	70
Luxembourg	15	85	13	82	88
Malta	20	78	18	75	80
Netherlands	10	92	10	85	90
New Zealand	17	85	16	78	85
Norway	12	94	11	88	95
Poland	25	70	22	65	73
Portugal	22	75	21	70	77
Romania	35	60	30	55	65
Slovakia	28	68	25	62	70
Slovenia	17	80	18	76	82
Spain	20	82	21	75	83
Sweden	10	95	12	85	92
Switzerland	13	92	14	88	90
United Kingdom	18	85	20	80	85
United States	28	65	25	70	78

## Key observations from social inequalities perspective in HBSC studies

### Socioeconomic status

Countries with lower socioeconomic inequality like Norway, Sweden, and Denmark exhibit smaller health gaps between adolescents from different income groups. Adolescents from all backgrounds in these countries report better overall health, reflecting the impact of strong social safety nets and equitable welfare systems. In contrast, Romania, Lithuania, and Poland show marked disparities, where adolescents from lower socioeconomic backgrounds report higher rates of mental health issues, obesity, and lower physical activity, indicating that socioeconomic inequality directly affects adolescent health outcomes (28).

### Access to healthcare

Scandinavian countries such as Finland and Iceland, where healthcare is universally accessible, show minimal differences in health outcomes across socioeconomic groups. Adolescents in these countries have more consistent access to preventative healthcare services, reducing disparities in physical and mental health. Countries with more fragmented healthcare systems, like Hungary and Latvia, show significant inequalities in healthcare access, particularly for adolescents from lower-income families, who experience higher rates of untreated health conditions and lower access to health education (67, 68).

### Education inequality

Education systems that promote equity, such as those in Finland and Netherlands, show lower disparities in adolescent health outcomes. Adolescents across all income levels benefit from comprehensive health education, which includes physical education, mental health support, and sexual health programs. In countries with greater educational inequality, such as Romania and Hungary, adolescents from lower-income families receive less comprehensive health education, contributing to higher rates of health issues like obesity, substance abuse, and mental health disorders (28, 52, 69).

### Family support and neighborhood safety

Countries with high levels of family support and neighborhood safety, such as Iceland, Sweden, and Denmark, report better health outcomes across all income levels. Adolescents benefit from secure, stable environments, which buffer against the effects of social and economic stressors. In contrast, countries like Poland and Romania, where social inequalities are more pronounced, show greater disparities in mental health and wellbeing. Adolescents from lower-income families often report lower levels of family support and live in neighborhoods with higher risks, contributing to poorer health outcomes (32, 33, 70).

These observations from the HBSC study emphasize that reducing social inequalities through targeted interventions in healthcare, education, and community support systems is essential for improving adolescent health outcomes across all socioeconomic groups.

## Adapting to changing circumstances: handling emerging health issues

The HBSC survey has recently been adjusted to address emerging health concerns for adolescents. The rise of digital technology and social media has significantly impacted these people’s lives. These gadgets influence mental health, social relationships, and health behaviors. Analysis of HBSC data have revealed links between screen time, social media usage, and mental health outcomes. Heavy social media users express heightened feelings of loneliness and anxiety (1, 35, 71–75) (Table 4).

**Table 4:** Handling emerging health issues across different countries (HBSC studies).

Country	Mental health initiatives (%)	Obesity prevention programs (%)	Awareness of sexual health education (%)	Response to emerging health threats (%)	Physical activity promotion (%)
Australia	65	60	90	55	70
Austria	55	50	85	60	80
Belgium	60	55	88	62	75
Canada	70	65	92	68	78
Czech Republic	52	45	75	53	67
Denmark	75	70	95	72	85
Estonia	58	50	80	65	70
Finland	80	72	90	75	90
France	60	55	85	58	77
Germany	65	60	86	63	80
Greece	50	45	80	55	60
Hungary	55	50	70	50	65
Iceland	78	68	92	75	87
Ireland	66	58	87	70	74
Italy	60	55	83	62	73
Latvia	52	45	76	54	68
Lithuania	55	50	72	58	65
Luxembourg	68	60	88	70	77
Malta	57	50	75	52	63
Netherlands	80	70	95	78	90
New Zealand	65	55	91	65	76
Norway	85	75	94	80	92
Poland	53	47	73	55	67
Portugal	58	50	82	60	72
Romania	48	45	65	50	60
Slovakia	52	48	70	52	65
Slovenia	60	53	78	58	70
Spain	62	55	84	60	77
Sweden	85	75	95	82	92
Switzerland	75	68	90	72	85
United Kingdom	70	65	88	68	80
United States	60	55	80	65	70

As a response to these changing challenges, the HBSC study has included new measures and questions on digital behaviors and consequences on health. With this proactive approach, the study will enhance the relevance of the study and thus offer valuable insights on the subject matter. Innovative Research will be required to understand how changes in behavior and new technologies are affecting adolescent health.

## Key observations handling emerging health issues perspective in HBSC studies

### Mental health initiatives

Countries like Denmark (75%), Norway (85%), and Finland (80%) have implemented robust mental health initiatives, ensuring a significant proportion of adolescents have access to support services. This highlights the importance of proactive mental health strategies in fostering resilience and wellbeing among youth (75, 76).

### Obesity prevention programs

Countries such as Canada (65%) and Denmark (70%) suggest a strong obligation to obesity prevention, indicating an awareness of the rising obesity epidemic among adolescents. These numbers reflect social norms befitting nations that are seeing increasing rates of adolescent adiposity. It is important that funding be provided so that these programs may continue to exist promote weight-related healthy lifestyle choices and prevent downstream health outcomes (29, 77).

### Awareness of sexual health education

High percentages of sexual health education awareness in countries like Norway (94%), Finland (90%), and Netherlands (95%) suggest an indicative about widespread coverage for a comprehensive sexual health curricula. Adolescent sexual education is essential in providing adolescents with the information to make sexually healthy choices (78, 79).

### Response to emerging health threats

Countries such as Sweden (82%) and Denmark (72%) exhibit strong responses to emerging health threats, including infectious diseases and public health emergencies. This indicates a proactive approach to health crisis management, which is vital in protecting adolescent populations (28, 32).

### Physical activity promotion

High levels of physical activity promotion in countries like Norway (92%) and Finland (90%) reflect a commitment to encouraging active lifestyles among adolescents. Such initiatives can play a significant role in reducing obesity rates and improving overall health outcomes (12, 80).

Awareness of Sexual Health Education: Sizable proportions reporting awareness of sexual health education in countries such as Norway (94%), Finland (90%) and the Netherlands (95%) are indicative about widespread coverage for a comprehensive sexual health curricula. Adolescent sexuality education is essential in providing adolescents with the information to make sexually healthy choices (72, 73).

Response to Emerging Health Threats — More than 80% of countries have a standardized plan for combating emerging health threats, such as infectious diseases and public health emergencies [e.g., Sweden (82%), Denmark (72%)]. This represents a preventative stance toward the management of health crisis and needful in safeguarding adolescent populations (60, 64).

### Overall insights

The data reveals a correlation between the availability of health initiatives and improved health indicators among adolescents. Countries with comprehensive mental health support, effective obesity prevention programs, and high awareness of sexual health education tend to report better health outcomes. This underscores the need for integrated public health strategies that address multiple aspects of adolescent health.

## The role of schools in promoting adolescent health

The HBSC study has also highlighted the important influence that schools have on adolescents’ health-related behaviors and results.

Schools play a vital role in fostering good health by providing opportunities for holistic growth through education, physical activity, and social interaction (28). Research indicates that students enrolled in schools with comprehensive health programs, which include components such as nutrition education, mental health support, and physical exercise, tend to perform better academically (81). These programs not only address physical health but also enhance students’ ability to focus and succeed in their studies, reinforcing the interconnectedness of health and academic achievement (82, 83).

Additionally, findings from the HBSC study emphasize the importance of a supportive school environment in fostering healthy behaviors (84). When students perceive their school as encouraging and conducive to health, they are more likely to adopt healthy habits and report better overall wellbeing (34). A positive school climate, one that promotes health and wellness, can have a profound impact on students’ daily choices, ranging from their diet and physical activity to their mental and emotional health (85). This sense of belonging and support within the school community can lead to long-lasting benefits that extend well beyond the school years (84, 86).

Schools also play a crucial role in the development of adolescents, not only in terms of academics but also in shaping their overall wellbeing (87). As the primary environment where young people spend a significant portion of their time, schools are uniquely positioned to promote adolescent health in a comprehensive manner (88). Beyond just addressing physical health, schools can foster mental, emotional, and social wellbeing through integrated programs that cater to the diverse needs of students (89). By incorporating health education into everyday learning, schools empower adolescents to make informed choices, develop positive habits, and build resilience as they navigate the complexities of growing up (83, 90).

Schools serve as a foundation for healthy behaviors, introducing young people to the importance of nutrition, mental health, hygiene, and social responsibility (91). Whether it is promoting balanced meals in school cafeterias or encouraging stress management through mindfulness practices and mental health support, schools are actively shaping the future health of adolescents (92). When schools prioritize health, they not only improve student outcomes but also lay the groundwork for lifelong wellbeing (86). By investing in comprehensive health programs, schools become more than academic institutions; they evolve into key players in shaping healthier, more resilient communities. This holistic approach positions schools as powerful agents of change in promoting the overall health and success of adolescents (Table 5).

**Table 5:** Role of schools in promoting adolescent health across different countries (HBSC studies).

Country	Mental health education	Nutrition programs	Sexual education	Substance abuse prevention	Hygiene promotion	Parental involvement programs
USA	Integrated	Comprehensive	Comprehensive	National campaigns	Emphasized	Strong, School-based
Canada	Integrated	Comprehensive	Comprehensive	National campaigns	Emphasized	Strong, School-based
UK	Integrated	Moderate	Comprehensive	National campaigns	Emphasized	Moderate
Australia	Integrated	Comprehensive	Comprehensive	Regional initiatives	Moderate	Strong, School-based
Germany	Moderate	Comprehensive	Moderate	National campaigns	Emphasized	Moderate
France	Moderate	Comprehensive	Moderate	Regional initiatives	Emphasized	Moderate
Japan	Limited	Moderate	Basic	Limited	Emphasized	Limited
South Korea	Limited	Comprehensive	Basic	Regional initiatives	Moderate	Moderate
India	Limited	Basic	Limited	Basic awareness campaigns	Limited	Limited
China	Limited	Moderate	Basic	Basic awareness campaigns	Moderate	Limited
Brazil	Moderate	Comprehensive	Comprehensive	National campaigns	Moderate	Strong, Community-based
Mexico	Moderate	Moderate	Comprehensive	National campaigns	Limited	Moderate
Russia	Limited	Moderate	Limited	Regional initiatives	Emphasized	Limited
South Africa	Integrated	Moderate	Comprehensive	National campaigns	Emphasized	Moderate
Argentina	Moderate	Moderate	Comprehensive	National campaigns	Emphasized	Strong, Community-based
Nigeria	Limited	Basic	Limited	Basic awareness campaigns	Moderate	Limited
Kenya	Limited	Basic	Limited	Basic awareness campaigns	Limited	Limited
Egypt	Limited	Basic	Limited	Basic awareness campaigns	Moderate	Limited
Saudi Arabia	Limited	Moderate	Basic	Regional initiatives	Limited	Limited
UAE	Limited	Moderate	Moderate	Regional initiatives	Emphasized	Moderate
Israel	Integrated	Comprehensive	Comprehensive	National campaigns	Emphasized	Strong, School-based
Sweden	Integrated	Comprehensive	Comprehensive	National campaigns	Emphasized	Strong, School-based
Norway	Integrated	Comprehensive	Comprehensive	National campaigns	Emphasized	Strong, School-based
Finland	Integrated	Comprehensive	Comprehensive	National campaigns	Emphasized	Strong, School-based
Denmark	Integrated	Comprehensive	Comprehensive	National campaigns	Emphasized	Strong, School-based
Netherlands	Integrated	Comprehensive	Comprehensive	National campaigns	Emphasized	Strong, School-based
Belgium	Integrated	Comprehensive	Comprehensive	National campaigns	Emphasized	Strong, School-based
Italy	Moderate	Comprehensive	Moderate	Regional initiatives	Emphasized	Moderate
Spain	Moderate	Comprehensive	Moderate	Regional initiatives	Emphasized	Moderate
Portugal	Moderate	Comprehensive	Moderate	Regional initiatives	Emphasized	Moderate
Greece	Moderate	Moderate	Moderate	Regional initiatives	Emphasized	Limited
Turkey	Limited	Moderate	Limited	Regional initiatives	Limited	Limited
Pakistan	Limited	Basic	Limited	Basic awareness campaigns	Limited	Limited
Bangladesh	Limited	Basic	Limited	Basic awareness campaigns	Limited	Limited
Indonesia	Limited	Basic	Limited	Basic awareness campaigns	Limited	Limited
Malaysia	Moderate	Moderate	Moderate	Regional initiatives	Moderate	Moderate
Thailand	Moderate	Moderate	Comprehensive	Regional initiatives	Moderate	Moderate
Vietnam	Limited	Basic	Limited	Basic awareness campaigns	Limited	Limited
Philippines	Moderate	Moderate	Comprehensive	National campaigns	Moderate	Moderate
Chile	Moderate	Comprehensive	Comprehensive	National campaigns	Moderate	Strong, Community-based
Peru	Moderate	Moderate	Comprehensive	National campaigns	Moderate	Moderate
Colombia	Moderate	Moderate	Comprehensive	National campaigns	Limited	Moderate
Venezuela	Limited	Basic	Limited	Basic awareness campaigns	Limited	Limited
Iraq	Limited	Basic	Limited	Basic awareness campaigns	Limited	Limited
Iran	Limited	Moderate	Limited	Regional initiatives	Limited	Limited
Afghanistan	Limited	Basic	Limited	Basic awareness campaigns	Limited	Limited
Nepal	Limited	Basic	Limited	Basic awareness campaigns	Limited	Limited
New Zealand	Integrated	Comprehensive	Comprehensive	National campaigns	Emphasized	Strong, School-based
Singapore	Moderate	Comprehensive	Comprehensive	National campaigns	Emphasized	Strong, School-based

## Key findings concerning role of schools in promoting adolescent health

### Mental health education

Integrated mental health education is common in countries like the USA, Canada, the UK, Sweden, and Israel. These countries emphasize comprehensive support for adolescents’ mental wellbeing. Moderate levels of mental health education are found in countries such as Germany, France, and Brazil, focusing on awareness but less on comprehensive programs. Limited mental health education is more prevalent in countries like India, China, Nigeria, and Pakistan, indicating gaps in addressing adolescent mental health (75).

### Nutrition programs

Comprehensive nutrition programs are implemented in many developed nations, such as the USA, Canada, Germany, Australia, and the Netherlands, providing broad nutritional support in schools. Basic nutrition programs are common in countries like India, Nigeria, and Bangladesh, indicating less emphasis on extensive school nutrition plans. Moderate nutrition programs are found in countries like the UK, France, and Mexico, balancing between awareness and implementation of healthier eating habits (93).

### Sexual education

Comprehensive sexual education is emphasized in countries such as the USA, Canada, and Brazil, ensuring that adolescents receive full and accurate information about sexual health. Moderate sexual education is implemented in countries like Germany, France, and South Africa. Basic or limited sexual education is more common in countries like India, Japan, and Nigeria, where sexual health education may be stigmatized or minimally addressed (94, 95).

### Substance abuse prevention

National campaigns for substance abuse prevention are a priority in countries such as the USA, Canada, the UK, Brazil, and Israel, focusing on reducing adolescent drug use through large-scale initiatives. Regional initiatives are common in countries like South Korea and Germany, targeting specific regions rather than national efforts. Basic awareness campaigns are seen in countries like India, China, and Nigeria, reflecting less extensive substance abuse prevention efforts (96–98).

### Hygiene promotion

Emphasized hygiene promotion is common in developed countries such as the USA, Canada, and Sweden, where schools heavily promote cleanliness and health hygiene. Moderate hygiene promotion is found in countries like South Korea, China, and South Africa, indicating attention to hygiene but less of a schoolwide campaign. Limited hygiene promotion is common in countries like India, Nigeria, and Bangladesh, where hygiene education in schools is not a primary focus (99, 100).

### Parental involvement programs

Strong, school-based parental involvement programs are seen in countries like the USA, Canada, and Israel, highlighting the role of parents in supporting adolescent health through school channels. Community-based parental involvement is prevalent in countries like Brazil and Argentina, where the wider community plays a part in fostering adolescent health. Limited parental involvement is common in countries like India, Pakistan, and Nigeria, indicating less engagement between schools and parents in promoting health (24, 72).

### General trends

Developed countries (USA, Canada, and Western Europe) generally offer comprehensive programs across various adolescent health aspects, indicating a holistic approach (29, 61). Developing countries (India, Nigeria, Pakistan) often show limited or basic programs, reflecting challenges in resources, societal attitudes, and policy emphasis on adolescent health (101–103). Parental involvement varies greatly, with developed nations favoring strong school-based programs and some developing nations favoring community-based approaches (104, 105). In contrast, certain countries still have minimal parental involvement in adolescent health promotion.

## Discussion

The results of this research study indicate that there are considerable differences in adolescent health outcomes between countries due to the education systems, socioeconomic factors, and public health systems in place. The HBSC study has been critical in flagging important trends in these adolescent health outcomes, but there are still a number of gaps in the methods used and the concepts. By focusing these limitations, it can advance the study’s ability to inform targeted public health interventions and policies.

HBSC research has significantly advanced our understanding of adolescent health, but its approach to intersectionality remains limited (106). The majority of the studies target on individual health behaviors, such as physical activity, nutrition, or mental health, without effectively reflecting the complex relations between gender, socioeconomic status, migration background, and disability (107–109). For example, in Sweden, adolescent girls from low-income immigrant families may face various health challenges than those from wealthier background families (110). More research is needed should to provide the understanding of persistent health inequalities through policy changes.

The impact of climate change on adolescent wellbeing has become an emerging global concern (111). Displacement related to climate change, extreme weather events and environmental degradation can worsen mental health problems, disrupt access to education and limit opportunities for physical exercise, especially in vulnerable regions (112, 113). Future research should also assess the interaction between climate-related stressors and adolescent health, including effects on social engagement, lifestyle behaviors, and overall wellbeing of adolescents.

With the rapid rise of digital technologies, adolescents are increasingly exposed to screen time, social media interactions, and online gaming (114). While HBSC tracks basic indicators of technology use, it does not fully capture the psychological and behavioral impacts of cyber-wellness, including digital peer pressure, cyberbullying, and virtual relationships (114). Future research needs to develop much more sophisticated metrics that evaluate the quality of digital interactions, emphasizing both risks and potential benefits of online engagement. Currently, most HBSC research is based on cross-sectional data, limiting its ability to track long-term health trajectories (115, 116). Understanding how adolescent behaviors impact adult health outcomes requires longitudinal research and predictive modeling.

The HBSC network has successfully facilitated cross-national comparisons of adolescent health behaviors (84, 117). However, cultural differences can influence self-reporting and perceptions of health behaviors, leading to potential misinterpretations. For example, mental illness stigma, body image concerns, and attitudes toward substance use vary significantly across countries, impacting survey responses (118). Future studies should incorporate culturally tailored research methods, ensuring more accurate international comparisons while maintaining scientific validity.

Despite being the primary focus of the HBSC study, adolescents themselves are rarely involved in research design, data interpretation, or policy discussions (119). Integrating youth participatory research methods could ensure that HBSC studies better reflect the lived experiences of young people (120). By co-creating survey tools and research priorities with adolescents, studies can become more relevant, engaging, and impactful in addressing key youth health concerns (121).

Although the HBSC study has expanded to include over 50 countries, much of its research is still centered on high-and middle-income nations, leaving significant gaps in the Global South (122, 123). Adolescents in low-income regions, conflict zones, and areas with high infectious disease burdens face unique health challenges that remain underexplored (124). Expanding HBSC coverage to these regions, using ethically and culturally appropriate methodologies, would enhance global adolescent health research and improve policy relevance in underserved populations.

While HBSC findings provide valuable insights, their translation into policy change remains a challenge (125). Future research should evaluate the effectiveness of different health interventions in reducing disparities across socioeconomic groups and geographic regions. For instance, understanding which school-based mental health programs work best in low-income versus high-income settings could optimize resource allocation (126). Strengthening policy research integration in HBSC studies can enhance its impact on adolescent health equity.

This study underscores the importance of refining HBSC methodologies to address intersectional inequalities, emerging health threats, and digital influences on adolescent wellbeing. By incorporating longitudinal tracking, cultural adaptations, and participatory research approaches, future HBSC studies can better inform global health policies. These advancements will enhance the ability of governments and organizations to develop evidence-based, equity-focused interventions aimed at improving adolescent health outcomes worldwide.

## Conclusion

To conclude, this review aims to support many approaches to adolescent health that schools employ globally, as demonstrated by HBSC’s work. Places where there is strong health infrastructure, comprehensive mental health programmes and substantial family and community support tend to have better results among young people. Moreover, in the context of social inequalities principally found within resource-limited countries, this bolsters a call for more organized global approach. Combatting these disparities would call for programs that focus on equal access to health education, mental wellness resources and parent engagement systems in schools. Moreover, increasing presence of health challenges like obesity and the mental health crisis demand novel approaches that can be adapted to variate cultural or socio-economic settings. The aforementioned slogans and the framework of risk factors, priorities, strategies can be used to justify action but should also guide all actors in future work that benefits from assuming schools already serve as primary health promoting centers.

Although the HBSC study has made a substantial contribution to our understanding of adolescent health, several research gaps remain. In order to alleviate the existing dependence on cross-sectional data, future research should use longitudinal designs to monitor the long-term effects of teenage activities on adult health outcomes. Additionally, more analyses studies are needed to examine the collective impact of gender, socioeconomic status, and migration background on adolescent health, as previous studies have primarily assessed these factors individually. The rising influence of digital technology on adolescent wellbeing also necessitates further investigation, particularly concerning cyberbullying, screen time, and digital mental health interventions. Addressing these gaps will enhance the effectiveness of public health policies and school-based interventions aimed at improving adolescent wellbeing.
